# Slum health: Diseases of neglected populations

**DOI:** 10.1186/1472-698X-7-2

**Published:** 2007-03-07

**Authors:** Lee W Riley, Albert I Ko, Alon Unger, Mitermayer G Reis

**Affiliations:** 1 Divisions of Infectious Disease and Epidemiology, School of Public Health, University of California, Berkeley, California, USA; 2 Division of International Medicine and Infectious Diseases, Weill Medical College of Cornell University, New York, New York, USA; 3 Gonçalo Moniz Research Center–Oswaldo Cruz Foundation (FIOCRUZ), Ministry of Health, Salvador, Bahia, Brazil; 4 School of Medicine, University of California-San Francisco, San Francisco, USA

## Abstract

**Background:**

Urban slums, like refugee communities, comprise a social cluster that engenders a distinct set of health problems. With 1 billion people currently estimated to live in such communities, this neglected population has become a major reservoir for a wide spectrum of health conditions that the formal health sector must deal with.

**Discussion:**

Unlike what occurs with refugee populations, the formal health sector becomes aware of the health problems of slum populations relatively late in the course of their illnesses. As such, the formal health sector inevitably deals with the severe and end-stage complications of these diseases at a substantially greater cost than what it costs to manage non-slum community populations. Because of the informal nature of slum settlements, and cultural, social, and behavioral factors unique to the slum populations, little is known about the spectrum, burden, and determinants of illnesses in these communities that give rise to these complications, especially of those diseases that are chronic but preventable. In this article, we discuss observations made in one slum community of 58,000 people in Salvador, the third largest city in Brazil, to highlight the existence of a spectrum and burden of chronic illnesses not likely to be detected by the formal sector health services until they result in complications or death. Lack of health-related data from slums could lead to inappropriate and unrealistic allocation of health care resources by the public and private providers. Similar misassumptions and misallocations are likely to exist in other nations with large urban slum populations.

**Summary:**

Continued neglect of ever-expanding urban slum populations in the world could inevitably lead to greater expenditure and diversion of health care resources to the management of end-stage complications of diseases that are preventable. A new approach to health assessment and characterization of social-cluster determinants of health in urban slums is urgently needed.

## Background

"Migrants from impoverished hinterlands, living without security, public health, and, often, clean water in the shantytowns of São Paulo, Lagos, Karachi, Dhaka, and Jakarta, have as much in common with each other as "People Like Us"–the global class of businessmen, journalists, academics, and anti-terrorism experts–do among themselves." –Pankaj Mishra, *Bombay: The Lower Depths*, New York Review of Books, November 18, 2004

**Figure 1:**
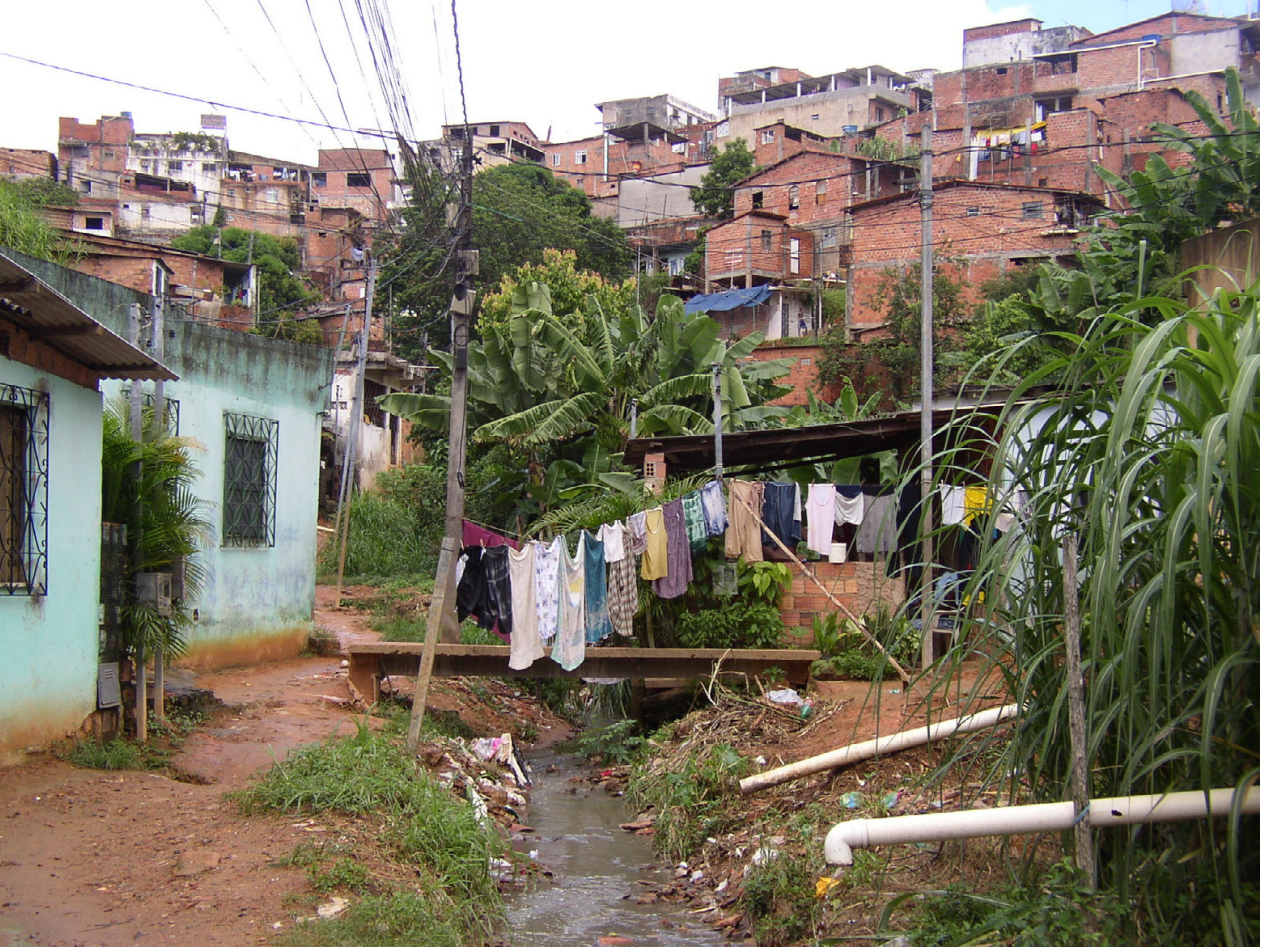
A *favela *in Salvador, Brazil.

**Figure 2:**
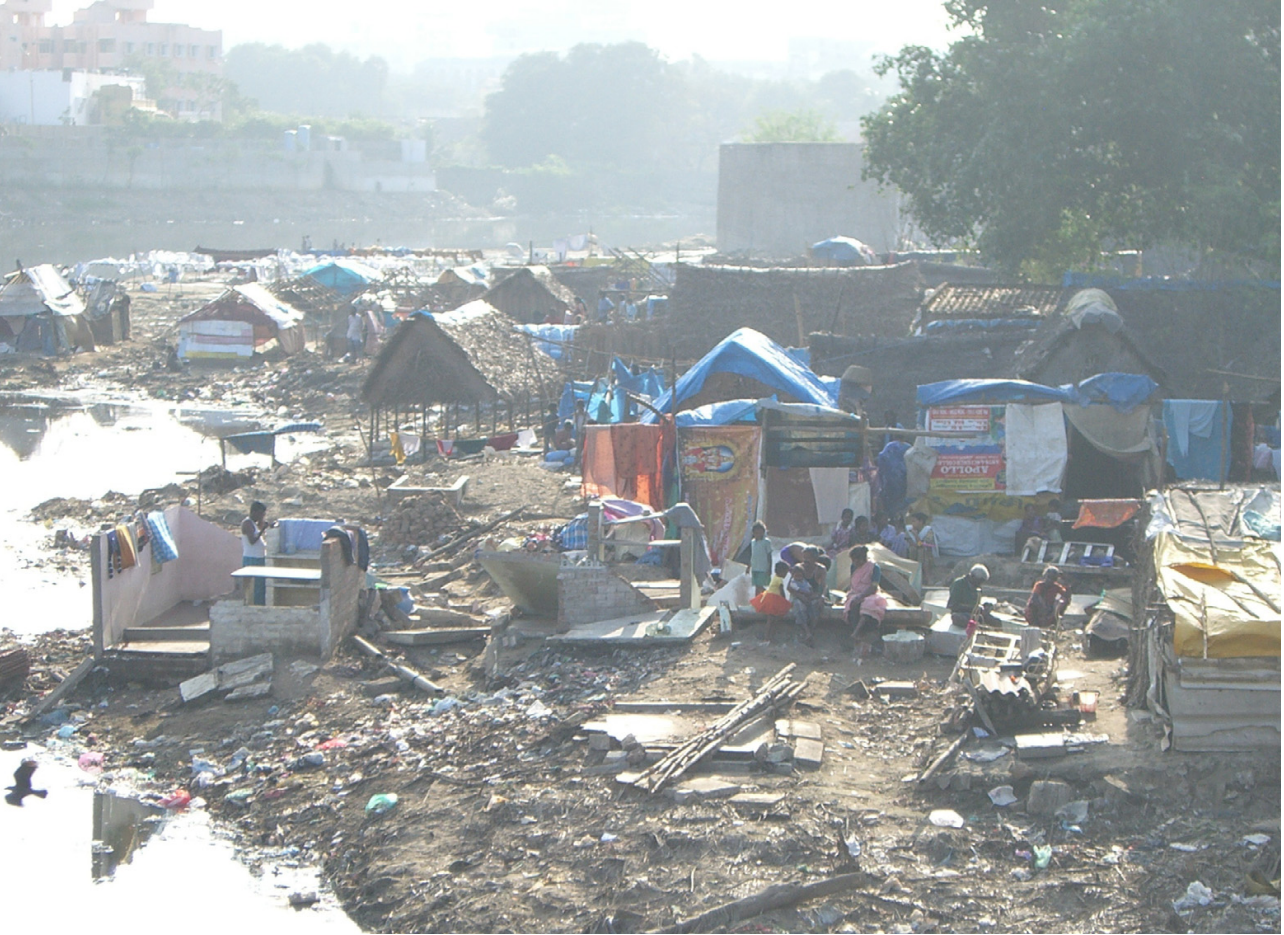
A *johpadpatti *in Chennai, India.

They are known as *favelas*, *kijiji*, *johpadpatti*, *gecekondu*, *aashiwa'i*, *barriadas*, *kampungs*, and *mudukku *(see Figures 1 and 2) [1]. They describe human settlements known in English as slums in Brazil, Kenya, India, Turkey, Egypt, Peru, Malaysia, and Sri Lanka, respectively [1]. In year 2000, the United Nations Millennium Declaration pledged to tackle the challenge of setting specific goals of achieving "significant improvement in the lives of at least 100 million slum dwellers by the year 2020" [2]. This historic declaration formally recognized the existence and need to improve the lives of a large group of people living in places in what are likely to become central to this century's most expensive health crisis. Today, nearly 1 billion people, or 32% of the world's urban population are estimated to live in slums [3]. In 30 years, this population is projected to increase to about 2 billion [3,4]. Thus, "achieving significant improvement in the lives of at least 100 million slum dwellers" in the next 13 years, if achieved, is not likely to make much of a dent in this global health challenge.

The United Nations Expert Group at a meeting held in Nairobi in 2002 operationally defined a slum as a human settlement that has the following characteristics: 1) inadequate access to safe water; 2) inadequate access to sanitation and other infrastructure; 3) poor structural quality of housing; 4) overcrowding; and 5) insecure residential status [3]. Currently, these characteristics describe communities that comprise 43% of the combined urban populations in all developing countries, and 78% of the urban population in least developed countries [3]. Thus, in many developing countries, life in slum settlements has already become the norm of urban human existence.

A recent report noted that 72% of the total global burden of disease in adults 30 years or older are due to chronic diseases [5]. In India and China, chronic diseases account for 53% and 80% of all nationally reported deaths, respectively [6,7]. These diseases receive the greatest amount of attention in developed countries or wealthy sectors of developing countries in terms of dollar amount spent for research, treatment, and prevention. We also know that education and prevention efforts for these diseases are effective and not costly [5,8]. These diseases are certainly not neglected. But what happens when they occur in neglected urban slum populations of the world? Here, we discuss how this social cluster–slums–creates health problems distinct to these communities that are not well recognized by the formal health sector, and how their further neglect could lead to economically disastrous consequences for nations with large urban slum populations.

## Discussion

Chronic non-communicable and communicable diseases like hypertension, diabetes, intentional and unintentional injuries, tuberculosis, rheumatic heart disease, and HIV infection are recognized to exist in slums because of the late complications of these diseases that the formal health sector sees and deals with. However, in slums, little is known about the magnitude, distribution, and risk factors for these illnesses before they manifest as stroke, myocardial infarction, kidney failure, suicide, multidrug-resistant TB, heart valve disease, and AIDS (see Table 1).

**Table 1:** Diseases that are chronic or associated with chronic conditions whose complications or end-stage outcomes require long-term medical intervention by the formal health sector services.

**Disease or condition**	**Complications or end-stage outcomes requiring formal health sector intervention**
**Chronic non-infectious diseases**	
Hypertension	Stroke; cardiovascular events, including myocardial infarction, congestive heart failure; kidney failure
Diabetes	Kidney failure requiring transplantation or dialysis; chronic infection (foot ulcer, osteomyelitis); acute recurrent infections (urinary tract infection, bacteremia, sepsis, pneumonia); blindness; sexual dysfunction
Asthma	Respiratory infection, respiratory failure
Ignored injuries (intentional or unintentional)	Chronic infection (osteomyelitis, non-healing wounds); limb deformity affecting ambulation, manual dexterity; long-term or permanent brain injury
Mental illnesses	Consequences of attempted suicide or homicide; violence; intractable behavior; restricted self-care
Reproductive health problems	Sterility; unwanted pregnancy; peripartum complications; congenital complications of infection (toxoplasmosis, CMV)

**Chronic infectious diseases**	
Tuberculosis, latent TB infection	Late-stage TB; Multidrug resistant TB
Hepatitis B, C	Liver cirrhosis; hepatocellular carcinoma
HIV infection	AIDS; opportunistic diseases

**Acute infectious disease with chronic outcomes**	
Sexually-transmitted infection	Reproductive diseases; AIDS
Skin lesion and superinfection	Bacterial superinfection; kidney failure due to post-streptococcal glomerulonephritis
Untreated bacterial pharyngitis; acute rheumatic fever	Post-streptococcal rheumatic heart disease requiring valve replacement

**Behavior and habits**	
Tobacco use	Cardiovascular diseases, cancer
Alcohol abuse	Liver failure, cirrhosis, unintentional injuries
Illicit drug use	HIV/AIDS; hepatitis B, C; endocarditis, unintentional injuries

In fact, it was not until the United Nations Human Settlements Program (UN-Habitat) report of 2003 that a comprehensive global description of slum communities became known [3]. The report describes in detail demographic, spatial, legal, economic, and social indicators of almost 1 billion people who satisfy the operational definition of slum dwellers. The report attempts to identify different approaches to address this problem to achieve the United Nations Millennium Declaration targets. It stresses participatory slum upgrading and poverty reduction programs. However, one major indicator not assessed in this global survey is health. Apart from the standard social indices, such as life expectancy at birth and under-five mortality rate, and access to improved water sources and sanitation, the report does not address disease spectrum or burden in these communities.

Because slum dwellers are intimately, if not formally linked economically, socially, and culturally to the rest of the urban population, the formal health sectors inevitably end up dealing with the consequences of the chronic diseases described above (Table 1). They deal with them at a tremendous cost to the national health system because they see these people only after they develop severe, near end-stage complications of the chronic diseases they have. For example, Brazil is estimated to expend $51,000,000 per year for surgical repair of heart valve diseases caused by the complications of a preventable disease–acute rheumatic fever–that occurs predominantly among slum children [9]. Those who receive an artificial valve then require life time care to prevent thromboembolism and infection, further contributing to health care cost. Many of these artificial valve recipients later require a second replacement of the valve. Little is known about the reservoir from which these cases arise or factors in the slums that lead to rheumatic heart diseases.

In fact, in many countries, most disease burden or mortality information on slum dwellers is largely based on clinic, hospital, or national mortality registry data. These end point data represent only the "tip of the iceberg". This type of information is not sufficient to plan health care expenditures, and grossly underestimates or misdirects the health care resource allocation needs.

We illustrate one example. In Salvador, the third largest city in Brazil, 60% of the 2.6 million inhabitants reside in slum communities defined by the census bureau as *favelas *[10]. One state infectious disease hospital is mandated to receive patient referrals from the city and its surroundings for all suspected cases of meningitis, leptospirosis, and meningococcal disease, regardless of their socioeconomic affiliation. Leptospirosis, transmitted by a spirochete excreted in rat urine, is a lethal disease (15% case fatality) in patients who present with severe manifestations such as acute renal failure, jaundice and pulmonary haemorrhage. Yet it is an entirely preventable disease. Active hospital-based surveillance initiated in 1996 found that the incidence of severe leptospirosis was 10 per 100,000 population. The major risk factor for acquiring severe manifestations of this disease was to be a resident of a *favela *[11]. More than 95% of these cases come from the city's various *favelas*. This observation led the investigators at the Oswaldo Cruz Foundation, Brazilian Ministry of Health to examine risk factors for this disease in one *favela *called Pau da Lima, a community of 58,000 people in Salvador (see Figure 1). A prospective cohort study found that the disease burden at the community level is significantly greater than that identified at the infectious disease referral hospital. Among inhabitants of this community, 5% are infected with the pathogenic spirochaete each year (unpublished results). Although all cohort subjects were *favela *residents, those who resided in close proximity to an open sewer were the major risk group for acquiring infection. These observations could not have been made by hospital-based or formal health sector-based disease surveillance.

Furthermore, in the process of performing this community-based study, the field team discovered that a large proportion of the residents of this shantytown suffered from a variety of chronic illnesses, both infectious and non-infectious. These included hypertension, diabetes, asthma, pyoderma secondary to scabies skin lesions, intentional and unintentional injuries and their chronic sequelae, mental illnesses, complications of drug and alcohol use, sexually-transmitted infections, and reproductive health problems. When community leaders of Pau da Lima were asked what they considered to be the most important health problems in their community, they reported hypertension, leptospirosis, and diabetes. They did not report diarrhea, tuberculosis, or acute respiratory infections–diseases frequently considered to be important in developing countries by developed country investigators. At the insistence of the community leaders, the Oswaldo Cruz Foundation performed a large hypertension screening survey among Pau da Lima residents and identified that hypertension rates (18% above 18 years of age) approached those observed in industrialized countries. More recently, obesity has become recognized in this population as a chronic condition. At this time, little is known about the burden of all the cases of stroke, congestive heart failure, kidney failure, rheumatic heart disease, and other complications that arise from this population in Salvador that the formal health care services manage.

In most cities of the world, slums, by definition, are informal and illegal settlements. A large proportion of the residents are rural migrants, displaced persons, illegal and legal immigrants, unemployed, and refugees. They are not necessarily all poor. There are those who are legally employed in formal sector occupations, such as school teachers and even university instructors, as well as those who are self-employed and own businesses [1]. However most are employed in low-paying occupations, such as in domestic services, the garment industry, solid waste recycling, security service, and day labor; even crime constitutes a type of income-generating activity for some residents [1,3]. Nearly all of these people are excluded from the usual benefits provided to or required for the formal sector employees, including minimal wage compensation, pension, let alone health insurance.

The world's urban population is increasing by about 70 million people a year [3]. These people require housing, employment, and services. The inability of governments in developing countries to meet their needs forces them to rely on or create their own informal infrastructure. Informal housing, informal employment, and self-employment are some of the survival activities adapted by these urban dwellers that are somewhat within their own power to implement. Over several decades, the largest *favela *in Rio de Janeiro called Rocinha has evolved from a squatter community predominantly consisting of wooden shacks, dirt and gravel streets and alleys, to a community with predominantly concrete and brick houses, paved streets, metered electricity, and piped water [1]. Even private banks and businesses have moved in [1]. Much of this transformation took place through the actions of community associations and other self-empowerment organizations that took advantage of their large population size as an economic incentive to lure in formal sector businesses and service providers [1]. Similar transformations have taken place in other shantytowns in major urban centers, especially in Asia and Latin America.

However, of all the basic human services available to slum dwellers, one that is beyond the control of these residents is health service. Health service, by definition, requires specialized, skilled, or trained personnel. It requires an infrastructure for delivery of care that involves provision of specialized information, physical examination, diagnostic services, hospitalization, medications, follow-up care, prevention, and surveillance. None of these services can be provided or created by the slum dwellers themselves. Furthermore, unlike electric or water companies, banks, or other private businesses, health service providers have little or no economic incentive to move into slums. Apart from those provided by volunteer groups, nongovernmental organizations (NGOs), and fee-for-service private clinics and pharmacies (usually run by unlicensed or poorly trained professionals or even nonprofessionals), health services are virtually nonexistent within most of the world's slums. Hence, health service is a social service that can only exist in the formal sector upon which the slum dwellers are completely dependent when they develop an end-stage disease.

While the world's governments worry and spend billions of health dollars in preparation for the uncertain Avian influenza pandemic and intentional release of pathogens, the world awaits a certain and unprecedented epidemic of chronic communicable and non-communicable diseases smoldering among burgeoning slum populations worldwide [4]. For emerging economy nations like Brazil, China, India, Thailand, and Mexico, this problem may reverse all the economic gains made in the last 2 decades, just as AIDS did to Africa in the last 20 years. Slum dwellers comprise a distinct urban population, whose health assessment and needs require novel approaches and focused solutions that cannot await the elimination of poverty and inequality [12,13]. While poverty reduction, self-empowerment, and elimination of disparity are important and worthy goals for improving health care in these communities, the speed of development and size of urban slums render achievement of these goals enormously challenging. Adequate and just characterization of the determinants of chronic and acute diseases in slums requires long-term prospective population-based surveillance. It also requires new science to understand social-cluster determinants of diseases. This is also challenging and expensive. But further neglect of this neglected population is likely to become even more costly.

## Summary

Little is known about the spectrum and burden of disease morbidity in urban slums of the world. The lack of such data hampers adequate health care resource allocation and provision of appropriate disease prevention services. The formal health sectors encounter slum dwellers only when they have end-stage complications of their chronic illness. They encounter these complications at a tremendous cost to their health care resources. Concerted effort is urgently needed to assess health burden and determinants of disease morbidity among slum residents at the community level.

## Competing interests

The author(s) declare that they have no competing interests.

## Authors' contributions

LWR and AIK formulated the original idea for this debate article. LWR wrote the initial draft, and AIK, AU, and MGR critically reviewed the manuscript and contributed additional ideas. All authors read and approved the final manuscript.

## Pre-publication history

The pre-publication history for this paper can be accessed here:


